# Towards integrated surveillance-response systems for the prevention of future pandemics

**DOI:** 10.1186/s40249-020-00757-5

**Published:** 2020-10-07

**Authors:** Jakob Zinsstag, Jürg Utzinger, Nicole Probst-Hensch, Lv Shan, Xiao-Nong Zhou

**Affiliations:** 1 Swiss Tropical and Public Health Institute, Basel, Switzerland; 2 University of Basel, Basel, Switzerland; 3 National Institute of Parasitic Diseases at the Chinese Center for Disease Control and Prevention & Chinese Center for Tropical Diseases Research, WHO Collaborating Center for Tropical Diseases, Shanghai, People’s Republic of China; 4 School of Global Health, Chinese Center for Tropical Diseases Research - Shanghai Jiao Tong University School of Medicine, Shanghai, People’s Republic of China

**Keywords:** Integrated surveillance-response, One health, Pandemics, Transdisciplinarity, Zoonoses

## Abstract

Most human pathogens originate from non-human hosts and certain pathogens persist in animal reservoirs. The transmission of such pathogens to humans may lead to self-sustaining chains of transmission. These pathogens represent the highest risk for future pandemics. For their prevention, the transmission over the species barrier — although rare — should, by all means, be avoided. In the current COVID-19 pandemic, surprisingly though, most of the current research concentrates on the control by drugs and vaccines, while comparatively little scientific inquiry focuses on future prevention. Already in 2012, the World Bank recommended to engage in a systemic One Health approach for zoonoses control, considering integrated surveillance-response and control of human and animal diseases for primarily economic reasons. First examples, like integrated West Nile virus surveillance in mosquitos, wild birds, horses and humans in Italy show evidence of financial savings from a closer cooperation of human and animal health sectors. Provided a zoonotic origin can be ascertained for the COVID-19 pandemic, integrated wildlife, domestic animal and humans disease surveillance-response may contribute to prevent future outbreaks. In conclusion, the earlier a zoonotic pathogen can be detected in the environment, in wildlife or in domestic animals; and the better human, animal and environmental surveillance communicate with each other to prevent an outbreak, the lower are the cumulative costs.

## Background

Most human pathogens originate from non-human hosts [1]. Diseases circulating between humans and animals are known as zoonoses, which are the key drivers of emergence and re-emergence of infectious diseases [2]. Zoonoses are classified into stages, depending on the modes of transmission and their epidemiology. Stage I pathogens are microbes present in animals but have not been detected in humans und natural conditions (Table 1). Stage II pathogens, such as brucellosis [4] and rabies [5], are continuously transmitted from animals to humans but are not transmitted between humans [3]. Successful elimination of stage II pathogens requires interventions in the animal reservoir [6, 7]. Stage III pathogens, such as monkey pox or *Leishmania donovani*, are transmitted to humans and lead to limited transmission that stutter to extinction. The basic reproductive number (*R*_0_), that is the number of secondary infections of one infected human, is below 1 in stage III pathogens. Stage IV pathogens persist in animal reservoirs. However, when they are transmitted to humans, it may lead to self-sustaining chains of transmission with *R*_0_ in excess of 1 [1]. It follows that stage IV pathogens represent the highest risk of future pandemics. For their prevention, the transmission over the species barrier — although rare — should, by all means, be avoided. Human immunodeficiency virus / Acquired immune deficiency syndrome (HIV/AIDS) and Corona virus disease-19 (COVID-19) are stage IV pathogens. Stage V pathogens are exclusive to humans, like malaria, measles, mumps or smallpox. In view of the devastating effects that HIV/AIDS and COVID-19 have not only on human health and wellbeing, but also the socio-cultural fabric and the economy, effective prevention of animal-human transmission cannot be overemphasized, as the financial resources very likely are much less than the primary and secondary (indirect) cost of a pandemic outbreak.

**Table 1:** Five stages leading to endemic human diseases (adapted and summarized from Wolfe et al. [3])

Stage	Description
I	Pathogens that are present in animals but have not been detected in humans und natural conditions.
II	Pathogens of animals that are transmitted from animals to humans, but are not transmitted between humans.
III	Pathogens of animals that are transmitted from animals to humans. In humans they don’t transmit easily and soon die out.
IV	Pathogens of animals that are transmitted to humans, leading to ongoing human to human transmission without the involvement of the animal host.
V	Pathogens that are exclusive to humans.

Transmission of a previously unknown coronavirus from animals to humans with subsequent global spread occurred in 2002 in the People’s Republic of China as severe acute respiratory syndrome (SARS) with masked palm civets as occasional direct source of human infection [8]. Later on, the transmission of coronavirus known as Middle Eastern respiratory syndrome (MERS) occurred in 2012 in Saudi Arabia with a likely origin in bats and camel intermediate host for transmission to humans [9]. COVID-19 is suspected to result from animal-to-human transmission of SARS coronavirus 2 (SARS-CoV-2) with most close relation to a bat coronavirus [10, 11]. The identification of origins and point sources remain still unclear and require further scientific inquiry to ascertain the zoonotic origin of COVID-19, especially on intermediary wildlife or domestic animal hosts.

Surprisingly though, most of the current research concentrates on the development and clinical testing of diagnostics, drugs and vaccines, while comparatively little research focuses on future prevention. The objective of this opinion paper is to explore options for the prevention of future pandemic infectious disease outbreaks with a focus on integrated One Health surveillance-response approaches.

**Fig. 1:**
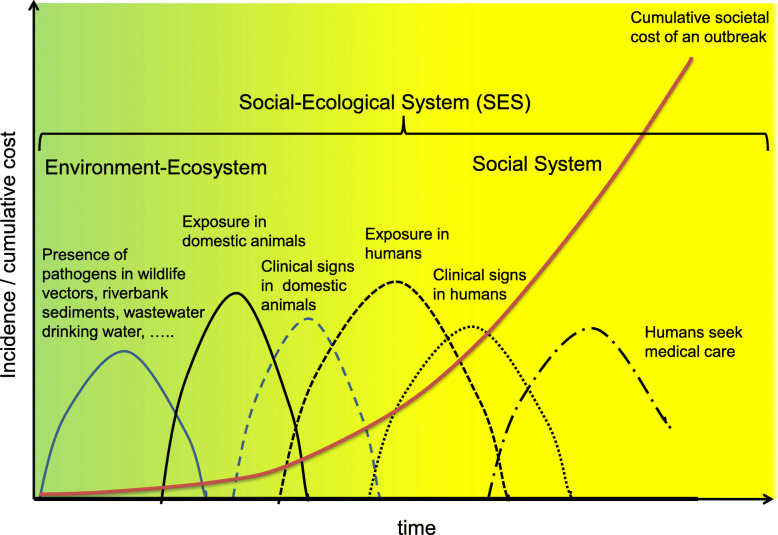
Schematic relationship of time to detection of an emerging pathogen and its cumulative cost of control. The changing green to yellow color represents the continuum of the environmental to the social system (adapted and expanded from [12, 22])

Already in 2012, the World Bank recommended to engage in a systemic approach for zoonoses control, considering integrated surveillance-response (iSR) and control of human and animal diseases for primarily economic reasons [12]. Integrated surveillance-response systems have been advocated in Africa [13] and have been promoted as part of the Field Epidemiology and Laboratory Training Programs (FETP/FELTP) [14, 15] and first integrated digital solutions like AfyaData have been proposed [16]. Bordier et al. present a comprehensive systematic review on existing integrated surveillance and response systems with a framework for their evaluation [17, 18]. These authors show that iSR systems are mostly used for West Nile virus, Rift Valley fever, influenza, schistosomiasis, zoonoses in general and for antimicrobial resistance surveillance. Despite these numerous examples [17, 19], contemporary surveillance-response systems remain separated for humans and animals [20]. Specifically, examples validating the postulated savings from iSR systems proposed by the World Bank are very rare. Pertaining to integrated West Nile virus (WNV) surveillance-response in mosquitos, wild birds, horses and humans, Paternoster and colleagues showed financial savings, when compared to single species surveillance in Emilia Romagna region, Italy between 2009 and 2015 [21]. WNV emergence in Europe is associated to climate change. In a recent study, integrated surveillance-response systems have been recommended to mitigate the effects of climate change [22]. A schematic proposed by the World Bank [12] has been extended to include also the environmental dimension, wildlife and waterbodies as potential reservoirs for zoonotic pathogens (Fig. 1). The general conclusion, however, remains the same as in the initial World Bank framework: (i) the earlier a zoonotic pathogen is detected in the environment, wildlife or domestic animals; and (ii) the better human, animal and environmental surveillance communicate with each other to prevent an outbreak, the lower are the cumulative costs.

The ongoing COVID-19 pandemic juxtaposes Fig. 1. Consequently, there is a pressing need to deepen the understanding of the interface or pathogen transmission between the environment, wildlife, domestic animals and humans as part of a complex social-ecological system (SES) [23, 24].

## Integrated surveillance-response systems

An integrated approach to environmental, animal and human health is termed One Health. In brief, One Health has been declared as a guiding principle for addressing the control of neglected tropical diseases and zoonoses by this open-access journal *Infectious Diseases of Poverty* [25]. Of note, One Health was described in the medical literature for the first time only in 2005 [26, 27]. One Health postulates the recognition and understanding of the inextricable linkage of humans and animals as a necessary but not sufficient requirement for a One Health approach. A sufficient requirement for a One Health approach is demonstrating an added value or synergistic benefit of a closer cooperation of human and veterinary medicine and related disciplines in terms of better health of humans and animals, financial savings or sustained environmental services when compared to the two medicines working in separation [28]. Successful examples of added value of One Health, which could not be achieved if human and animal health sectors work separately, have been reported and are summarised in Table 2 [22].

**Table 2:** Examples of added value of One Health, compared to separated human and animal health approaches (adapted from [22, 29])

Domain	Added value
Health services	Joint human and animal vaccination services for mobile pastoralists in Chad provide access to health care for populations, which would otherwise be excluded, and hence, financial and human resources can be saved.
Brucellosis control	Mass vaccination of livestock against brucellosis in Mongolia does not only benefit public health, but is approximately three times more profitable from a societal perspective.
Rabies control	Dog mass vaccination and human post-exposure prophylaxis in Chad is less costly than human PEP after about 10 years.
Schistosomiasis control	An integrated control strategy, facilitated by intersectoral cooperation (e.g. Ministry of Agriculture, Ministry of Forestry, Ministry of Health and Ministry of Water Conservation) has accelerated the programme for schistosomiasis elimination in the People’s Republic of China in more than 90% of the endemic areas.
Surveillance and response	Integrated surveillance-response of WNV in the Emilia Romagna region, Italy, saved more than one million Euro in the period of 2009–2015, compared to separate human and animal surveillance.
Infrastructure	The Canadian Science Centre in Winnipeg, hosting laboratories under one roof for highly contagious diseases affecting humans and animals alike saves 26% of the operations cost, compared to two separate human and animal health laboratories.
Communication	A recent outbreak of Q-fever in the Netherlands with several thousand human cases could probably have been avoided if the veterinary and public health authorities had maintained continuous communication.

*WNV* West Nile Virus; *PEP* Post-exposure prophylaxis

## An integrated systemic approach to prevent future pandemics

Already the first scholarly paper introducing the concept of One Health in 2005 stated, with regard to avian influenza, that: “research for [ …] vaccines should urgently be complemented by modifications to smallholder livestock systems and live-animal markets to prevent or reduce interactions between [wildlife] and [livestock], which might be reservoirs for future human [ …] pandemics” [26]. “However, these implementations should be handled carefully to avoid impeding poverty.” This warning, published 15 years ago in *The Lancet*, sounding like a forecast in face of the current COVID-19 pandemic, remained largely unheard. A study pertaining to an integrated systemic approach for schistosomiasis control in Yunnan Province, China, coupled with systems modelling, showed that the approach integrated with ecological management was able to accelerate the implemented in schistosomiasis endemic area resulting in an improvement of the co-effectiveness of environmental protection and schistosomiasis control [30].

**Fig. 2:**
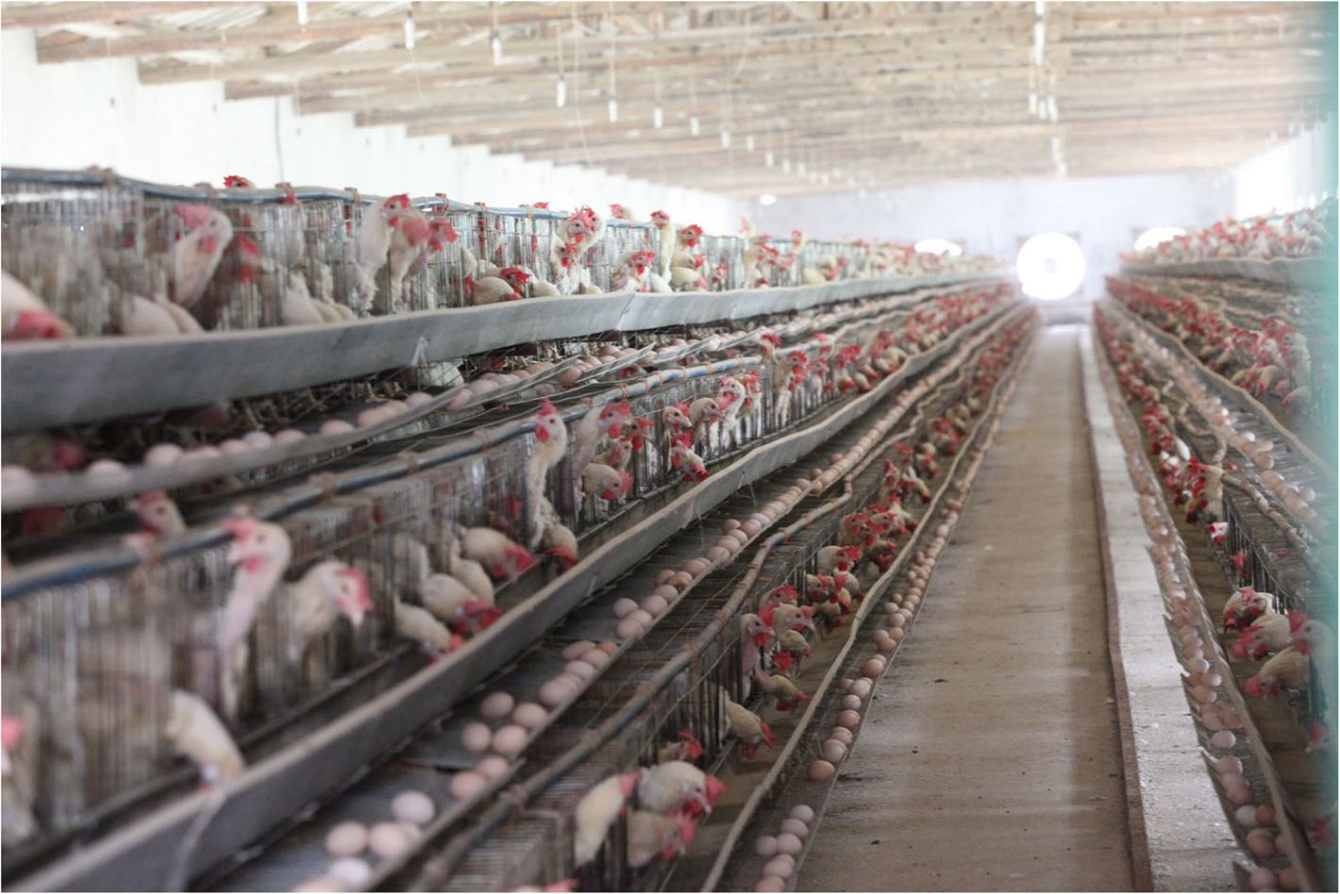
Intensive and subsistence livestock production are an income source for hundreds of millions of small-holder farmers but also exposing animals and humans to infectious disease

We urgently need to investigate the biosecurity of live animal markets, intensively farmed chickens or pigs, and other interfaces of multiple animal (wildlife and domestic species) [31]. To improve biosecurity in live animal markets and on farms, animal welfare needs to be fundamentally changed, as animals are often kept under inacceptable humane standards (Fig. 2) and very poor sanitary conditions. At the same time, domestic animal husbandry contributes to the livelihoods of hundreds of millions of small farmers. Drastic measures can lead to the loss of income and impeding poverty and hunger for large numbers of small-scale famers. For this reason, all stakeholders (e.g. farmers, traders, butchers, consumers, administrators and scientists) should be involved to identify locally adapted biosecurity and animal welfare measures, while maintaining economic activity.

## Transdisciplinary participatory approaches

National states are in a normative dilemma of preventing new outbreaks at the cost of economic hardship for millions of small-scale farmers and other economic actors. Governments and experts alone cannot solve this dilemma. All actors having their stakes need to be involved in a societal consensus to jointly enhance biosecurity, without compromising economic activities. What a nation is prepared to engage in the prevention of new outbreaks through better biosecurity has to be negotiated in every context independently. There are no blueprints, but the engagement of academic and non-academic actors in a transdisciplinary process has a high potential to find locally adapted solutions, as recommended by the Organization for Economic Co-operation and Development (OECD) [32]. As evidenced by the COVID-19 pandemic, efforts have to move even beyond combining the surveillance of human and animal infections, but to also to integrate the surveillance of non-communicable diseases. Furthermore, without citizen engagement and a specific focus on the social sciences, it will not be possible to achieve broad acceptance of integrated surveillance-response measures. The COVID-19 pandemic is full proof of the transdisciplinary breadth required.

In summary, the biosecurity of the wildlife-domestic-animal interface must be improved to reduce the risk of zoonotic transmission of diseases with pandemic potential [11]. iSR systems, involving environmental, wildlife, domestic animals and humans can reduce the time to detection of new emerging disease outbreaks and to safe financial resources. Response mechanisms should include a broad spectrum of concerned stakeholders to manage in the same time the spread of disease and to avoid economic decline.

## Conclusions

To prevent future pandemics like COVID-19, there is a pressing need to deepen the understanding of the interface or pathogen transmission between the environment, wildlife, domestic animals and humans as part of a complex social-ecological system. For this, integrated environment-wildlife-livestock-human surveillance-response system have a high potential because the earlier a zoonotic pathogen is detected in the environment, wildlife or domestic animals; and the better human, animal and environmental surveillance communicate with each other to prevent an outbreak, the lower are the cumulative costs.

Stakeholders (e.g. farmers, traders, butchers, consumers, administrators and scientists) should be involved to identify locally adapted biosecurity and animal welfare measures, while maintaining economic activity. A broad acceptance of integrated surveillance-response measures requires citizen engagement and a specific focus on the social sciences.

## Data Availability

Not applicable.

## Abbreviations

COVID-19: Coronavirus disease 2019
MERS: Middle Eastern respiratory syndrome
OECD: Organization for Economic Co-operation and Development
PEP: Post-exposure prophylaxis
*R*_0_: Basic reproductive number
SARS: Severe acute respiratory syndrome
SARS-CoV-2: Severe acute respiratory syndrome coronavirus 2
SES: Social-ecological system
WNV: West Nile Virus
